# Neutralization of Japanese Encephalitis Virus by heme-induced broadly reactive human monoclonal antibody

**DOI:** 10.1038/srep16248

**Published:** 2015-11-06

**Authors:** Nimesh Gupta, Mélissanne de Wispelaere, Maxime Lecerf, Manjula Kalia, Tobias Scheel, Sudhanshu Vrati, Claudia Berek, Srinivas V. Kaveri, Philippe Desprès, Sébastien Lacroix-Desmazes, Jordan D. Dimitrov

**Affiliations:** 1 INSERM, UMR S 1138, Centre de Recherche des Cordeliers, Paris, France; 2 Université Pierre et Marie Curie-Paris6, UMR S 1138, Paris, France; 3 Université Paris Descartes, UMR S 1138, Paris, France; 4 Flavivirus-Host Molecular Interactions laboratory, Virology Department, Institut Pasteur, Paris, France; 5 Vaccine and Infectious Disease Research Centre, Translational Health Science and Technology Institute, Faridabad, Haryana, India; 6 Deutsches Rheuma-Forschungszentrum, Institut der Leibniz-Gemeinschaft, 10117 Berlin, Germany; 7 Infection and Epidemiology Department, Institut Pasteur, 75724 Paris & UMR PIMIT (I2T), University of Reunion Island, INSERM U1187, CNRS 9192, IRD 249, GIP-CYROI, la Reunion, France

## Abstract

Geographical expansion and re-emerging new genotypes of the Japanese encephalitis virus (JEV) require the development of novel therapeutic approaches. Here, we studied a non-conventional approach for antibody therapy and show that, upon exposure to heme, a fraction of natural human immunoglobulins acquires high-affinity reactivity with the antigenic domain-III of JEV E glycoprotein. These JEV-reactive antibodies exhibited neutralizing activity against recently dominant JEV genotypes. This study opens new therapeutic options for Japanese encephalitis.

The appearance of new highly virulent genotypes of JEV is a growing cause of concern^1^,^2^. Japanese encephalitis (JE) is a vaccine-preventable disease, however, due to its enzootic transmission it can never be eradicated from the natural environment. Therefore, even though vaccination can reduce its incidence, effective antiviral therapy is a necessity to supplement existing strategies for controlling the disease^3^.

Following an infected mosquito bite, the virus replicates at low levels in the spleen and spreads by haematogenous route to other parts of the body including the central nervous system. In this early phase, the immune response efficiency determines disease outcome^4^. Although both the humoral and cellular arms of the immune system are involved in immunity to JEV, their relative contribution is not well understood. Importantly, failure to efficiently produce virus-specific antibodies (Abs) is associated with an increased likelihood of developing severe disease^5^. Indeed, the passive transfer of neutralizing Abs was shown to protect mice against JEV infection^6^,^7^,^8^,^9^,^10^.

In animals, the administration of neutralizing Abs is mostly efficient when delivered at the same time as the virus challenge. In humans, JE has an incubation period of 5 to 15 days and non-specific symptoms may last for up to 6 days. It is therefore uncertain whether the mere targeting of the virus using JEV-specific Abs would be therapeutically efficient. In fact, an intravenous IgG preparation (IVIg) that is not hyper-immune to JEV showed therapeutic benefits in the recovery from JE^11^. The protection conferred by IVIg was attributed to its anti-inflammatory potential^12^. Indeed, hyper-inflammation at the periphery or in the brain plays a decisive role in JEV pathogenesis^13^,^14^,^15^,^16^. Hence, the ideal Ab candidate for passive therapy should target both the virus as well as the associated hyper-inflammation.

In all healthy individuals a fraction of Abs can be detected that acquire novel antigen-binding specificities or polyreactivity upon *in vitro* or *in vivo* exposure to redox agents, including the ubiquitous cofactor molecule heme^17^,^18^,^19^. Notably, heme was found to confer novel binding specificities to Abs without influencing binding to their cognate antigen^19^,^20^. Importantly, the contact of Abs with redox agents also results in a substantial increase of their anti-inflammatory potential. Thus, heme or ferrous ions exposed human immunoglobulins considerably improved the survival in mouse model of bacterial sepsis and inhibited the development of autoimmune inflammation of insulin islet cells in mice^18^,^21^. Therefore, Abs with inducible polyreactivity may represent an appropriate therapeutic tool for JEV-mediated disease.

## Results and Discussion

**Figure 1:**
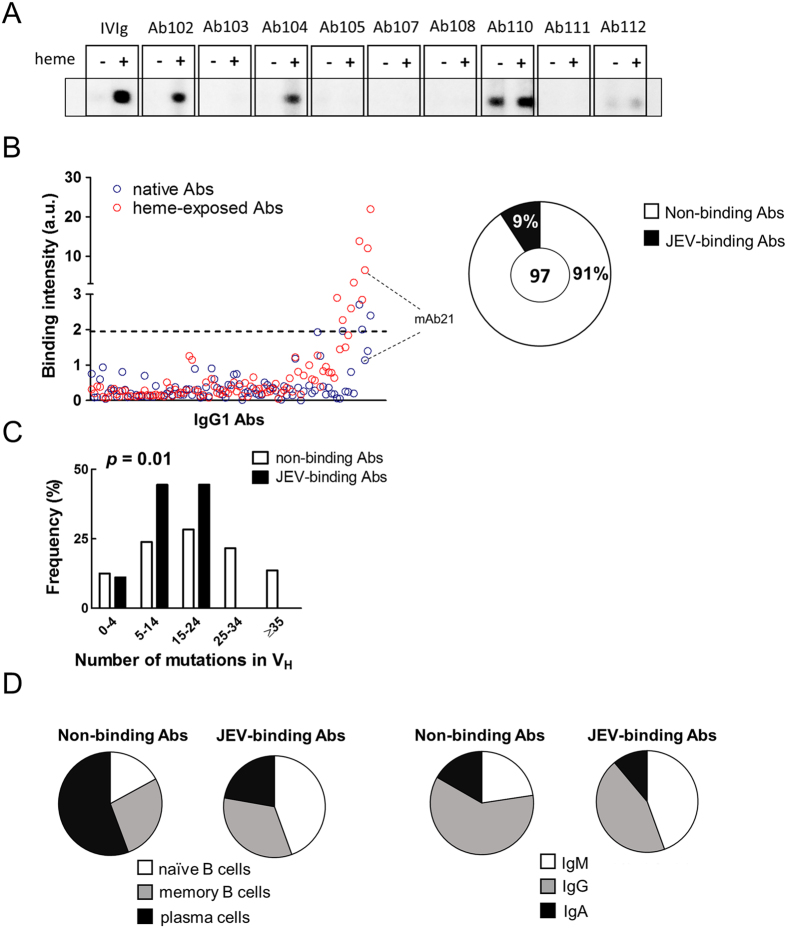
Repertoire analyses of prevalence, origin and characteristics of heme-induced JEV binding Abs. (**A**) Representative data obtained by immunoblot analysis of interaction of different native and heme-exposed human Abs with immobilized JEV E protein. The JEV protein was directly immobilized on the surface of nitrocellulose membrane. Each monoclonal Ab was diluted to 20 μg/ml and incubated with the immobilized JEV E. As controls, native and heme-exposed pooled human IgG (IVIg) were used. (**B**) Binding intensity of native and heme-exposed human IgG1 to JEV E. The plot depicts the reactivity to JEV E of 97 human monoclonal IgG1 Abs. Each point represents the binding intensity of a particular native (blue circles) and heme-treated (red circles) Ab. Binding intensity were calculated by densitometric analyses of immunoblots after subtraction of background binding to nitrocellulose membranes. The dashed line, defined by the sum of the average binding intensity of all native Abs plus three standard deviations, shows the threshold of positivity. The pie graph depicts the fraction of Abs that acquires JEV E specificity after heme exposure (full area), and the fraction that displays no change in reactivity (empty area). (**C**) Frequency distribution analyses of number of somatic mutations in VH regions of Abs that gain JEV E reactivity and Abs insensitive to heme exposure. The Fisher’s exact test was applied to evaluate the statistical significance of the frequency distributions. (**D**) The pie graphs depict the fraction of B cell subpopulations or the distribution of percentages of original B cell receptor isotype from heme insensitive (left pie chart) or sensitive (right pie chart) Abs. Statistical significance was assessed by using Fisher’s exact test.

To test this hypothesis, the frequency of Abs that acquire specificity to JEV proteins upon exposure to heme in the human immune repertoire was determined. We analyzed a panel of 97 human recombinant monoclonal IgG1, cloned from different subpopulations of B cells isolated from the synovial tissue of patients with rheumatoid arthritis^22^. Following exposure to heme, approximately 9% of Abs acquired binding specificity to the JEV envelope (E) protein (Fig. 1A,B). Interestingly, while analyzing the same repertoire, we observed that 24% of the Abs acquire reactivity to HIV-1 gp120^23^. Among JEV E binding Abs all gained reactivity to gp120 as well. Future studies may decipher whether this difference in the frequencies of heme-induced Abs is due to the different mechanisms underlying heme-induced specificity, or differential characteristics of the viral antigens. Further analyses of the characteristics of the variable region sequences of the immunoglobulins revealed that Abs, that acquired reactivity towards JEV E upon heme exposure have significantly lower number of somatic mutations (Fisher’s exact test *P* = 0.01) (Fig. 1C). Most of the Abs gaining reactivity to JEV E protein originated from naïve B cells (comparison of frequency of plasma and naïve B cells by Fisher’s exact test *P* = 0.04) and thus expressed IgM as original isotype (Fig. 1D). The gene families encoding the variable regions genes, the length of CDR3s and the prevalence of different amino acids in the CDRs of the light and heavy chains of Abs were similar whether Abs acquired binding to JEV E or not upon exposure to heme (Fig. S1).

**Figure 2:**
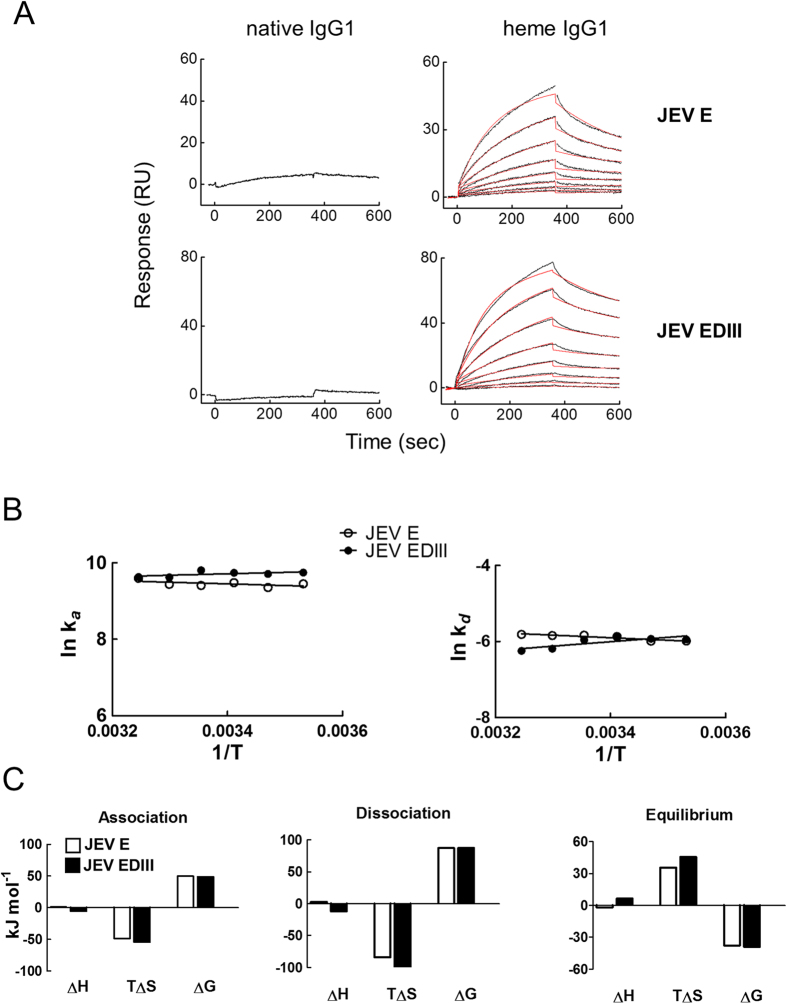
Kinetic and thermodynamic analyses of interaction of heme-exposed human IgG1 (mAb21) with JEV E and EDIII proteins. (**A**) Real-time interaction profiles of binding of native or heme-exposed human monoclonal IgG1, mAb21 to immobilized recombinant JEV E and EDIII proteins. The real-time interaction profiles obtained after injection of native mAb21, diluted to 500 nM are presented in the left panels. The binding profiles of heme-exposed mAb21 at 500, 250, 125, 62.5, 31.25, 15.63, 7.81, and 3.90 nM are presented on the right panels. The binding analyses were performed at 25 °C. The graphs show experimentally determined binding curves (black lines) and curves generated by globally fitting the data by BIA evaluation software (red line). The estimated kinetic parameters are presented on Table 1. (**B**) Arrhenius plots showing the natural logarithm values of association and dissociation rate constants of the heme-sensitive mAb21 obtained after interaction with JEV E (open circles) and JEV EDIII (filled circles) as a function of reciprocal values of temperature (in Kelvins). To generate these plots the kinetic rate constants were determined by global analysis of sensorgrams generated after evaluation of binding kinetics of the heme-exposed mAb21 with immobilized JEV proteins at varying temperatures (10, 15, 20, 25, 30, and 35 °C). Linear regression analyses were applied to obtain the slopes of the temperature dependency. (**C**) Association, dissociation and equilibrium thermodynamic parameters of binding of heme-exposed mAb21 to JEV E and EDIII. Changes in the enthalpy, entropy and free energy during different phases of the interaction of heme-exposed mAb21 with JEV E (white bars) and EDIII (black bars) are depicted. The changes in non-equilibrium thermodynamic parameters were evaluated by applying Eyring’s analyses on the data from Arrhenius plots.

To delineate the mechanistic and functional understanding of induced JEV recognition, we focused on a monoclonal IgG1 (mAb21) that demonstrated high sensitivity to heme-induced specificity for JEV (Fig. 1b). First, mAb21 (1 μM) was exposed to hematin (5 μM) and the binding to JEV E and to the recombinant domain-III^24^ of JEV E (EDIII, Fig. 2A) was studied. Kinetic analyses demonstrated that exposure of initially non-reactive mAb21 to heme resulted in acquisition of binding affinity in the nanomolar range to both, JEV E and EDIII (Table 1). As a reference, the JEV E-reactive mouse monoclonal Ab 4G2 (ATCC HB-112) also displayed affinity in nanomolar range to JEV E (Fig. S2). Moreover, thermodynamic analyses revealed that recognition of JEV E and EDIII by heme-exposed mAb21 is an entropy-driven process with minimal contribution of enthalpy (Fig. 2B). The negative values of association entropy imply that interaction of the heme-exposed mAb21 with both JEV proteins is accompanied by structural reorganizations or by changes in the solvent structure (Fig. 2C). For further biological characterizations, we also studied a therapeutic monoclonal IgG1 (Rituxan; Rtx) that has already been described to acquire substantial antigen-binding polyreactivity after exposure to heme^25^. The thermodynamic mechanism of recognition of JEV proteins by both heme-exposed Rtx and mAb21 were similar (Table 1, Fig. S3A).

**Figure 3:**
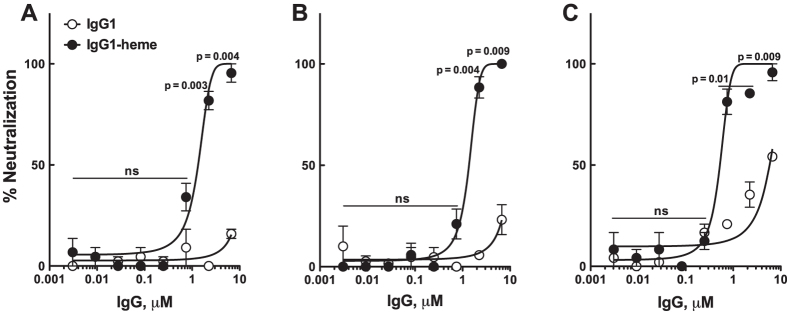
*In vitro* neutralization of JEV by heme-treated human monoclonal IgG1. Neutralization potential of JEV-reactive heme-exposed IgG1 (Rtx) was accessed on distinct genotypes of JEV selected on the basis of their recent circulation in Southeast Asia. Neutralization titrations by focus-reduction neutralization test (FRNT) using either heme-treated or native Rtx are shown. **(A)** FRNT on a genotype III strain (JEV-RP-9)^29^ **(B)** FRNT on a chimeric virus that express the structural proteins of a genotype I strain (CNS769_Laos_2009)^30^ and the nonstructural proteins of a genotype III strain (JEV-RP-9). **(C)** FRNT on a genotype V strain (JEV-XZ0934)^29^. The Y-axes depict the percent neutralization that was calculated using the formula: 100 × (1-FFU of treatment/FFU of control). Results are depicted as means ± SEM of two independent experiments performed in duplicate for each concentration. Statistical significance of the differences between heme-treated or native IgG1 was assessed at each concentration using two-tailed unpaired Student’s t-test. ns: non-significant.

Finally, we evaluated the functional efficacy of the heme-induced JEV-reactive Abs in a focus-reduction neutralization test (FRNT) on BHK-21 cells. First Rtx (33.5 μM) was exposed to hematin (50 μM). The neutralization potency was then assayed using 3-fold serial dilutions of the heme-exposed Ab. The JEV-reactive Ab demonstrated a dose-dependent neutralization of JEV GIII with an absolute IC_50_ of 1.33 ± 0.12 μM (heme-treated over native: *P* = 0.004 at 6.7 μM; *P* = 0.003 at 2.2 μM; Fig. 3A). It also demonstrated broad neutralization capacity since the induced JEV-reactive Ab also neutralized the highly pathogenic and dominating JEV genotypes GI with an absolute IC_50_ of 1.36 ± 0.09 μM (heme-treated over native: *P* = 0.009 at 6.7 μM; *P* = 0.004 at 2.2 μM; Fig. 3B) and GV^26^ with an absolute IC_50_ of 0.52 ± 0.04 μM (heme-treated over native: *P* = 0.009 at 6.7 μM; *P* = 0.01 at 2.2 μM; *P* = 0.01 at 0.7 μM; Fig. 3C). Whether this broad reactivity is the result of higher epitope accessibility on the viron envelope or due to the Ab docking on particular epitope in EDIII domain remains to be explored^27^. Controls that consisted of heme alone or heme-exposed albumin had no neutralizing effect on the virus infectivity, indicating that heme alone does not have direct effects on reducing virus infectivity in our experimental setup.

Our study highlights the prevalence and the binding mechanism of heme-inducible JEV reactive Abs in the human immune repertoire. Furthermore, our data suggest the biological potency of these Abs in neutralizing distinct JEV genotypes. Thus, our approach may be of value in producing therapeutic Abs of new defined specificity from the normal human immune repertoire. Although previously validated in animal models, the anti-inflammatory effects of heme-inducible antibody remains to be explored in JE infection model. Moreover, simultaneous to these validations, conferring the anti-inflammatory potential to existing JEV-neutralizing Abs by exposure to redox agent will also be appealing to identify a novel candidate therapy. Additional in depth investigations in suitable animal models are thus warranted to establish the value of our approach.

## Methods

### Recombinant proteins and antibodies

The domain III (EDIII) of JEV envelope protein cloned from an Indian strain (Vellore P20778) of JEV genotype III was synthesized in E.coli as explained elsewhere^24^. The recombinant envelope (E) protein (MBS143155) was originally cloned from JEV genotype III (Kamiyama strain) and obtained commercially from MyBioSource, San Diego, CA, USA.

Details about the generation of the repertoire of human monoclonal IgG1 antibodies were provided elsewhere^22^,^28^. Briefly, the variable genes encoding the immunoglobulin heavy and light chains were amplified from different B cells subpopulations by single-cell PCR from synovial tissue of rheumatoid arthritis patients, cloned in PUC19 vector containing the genes encoding the constant Fc-γ1 or λ/κ chains, respectively, and expressed using HEK293. Although the panel of antibodies was generated from patients with autoimmune disease, studied antibodies did not expressed biased tendency for binding to self-antigens. In some cases therapeutic humanized IgG1 antibody Rituxan (Roche, Basel Switzerland) was used.

### Reagents

Stock solutions of oxidized *heme b* (ferriprotoporphyrin IX) were prepared by dissolving hemin (Fluka, St. Louis, USA) in 0.05N solution of NaOH. The heme stock solution was kept at 4 °C. The treatment of immunoglobulins was always performed with freshly prepared heme, at dim light conditions.

### Screening of repertoire of human IgG1 for gain of reactivity to JEV E protein after exposure to heme

JEV E protein was diluted to 20 μg/mL in PBS (pH 7.4) and immobilized on nitrocellulose membranes using 28 channel Miniblot system (Immunetics, Boston, USA). The nitrocellulose membrane was incubated overnight at 4 °C. After immobilization, the membranes were blocked by incubation at 25 °C for 1 hour in TBS buffer containing 0.1% of Tween-20. After blocking the membranes were again mounted in Miniblot apparatus in a direction perpendicular to one used for immobilization of the proteins. Recombinant human antibodies were exposed at a 40 μg/mL concentration, either with hemin solubilized in 0.05N NaOH or with vehicle only. The antibodies were treated with final heme concentration of 20 μM. After a 30 min incubation on ice, heme-treated and native recombinant antibodies were diluted in TBS containing 0.1% Tween 20 (TBS-T) to final concentration of 20 μg/ml and incubated for one hour on the membrane containing immobilized JEV E protein. As a positive control, 10 μM (1.5 mg/ml) pooled human IgG (IVIg) was treated with hemin (20 μM) and incubated with immobilized proteins at final IgG concentration of 0.05 μM (7.5 μg/ml) in TBS-T. After washing for one-hour membrane was incubated with anti-human IgG antibody conjugated with HRP, clone JDC-10 (Southern Biotech, Birmingham, Alabama, USA). Immunoreactivities were revealed by using Pierce ECL western blotting substrate (Thermo Scientific, Rockford, USA) and chemiluminescence signal was recorded on multipurpose film (GE Healthcare, Little Chalfont, UK).

Intensities of spots obtained at the intersections of JEV E protein and antibodies-loaded channels were evaluated by densitometry using ChemiCapt/Bio1D software (Vilber Lormat, Torcy, France). The background signal obtained after incubation of studied antibodies with nitrocellulose membrane alone was always subtracted from signal obtained in the presence of immobilized protein. Values were normalized to controls present on each membrane to account for variations in exposure times. To identify the antibodies that gain significant binding to JEV E proteins after exposure to heme, values of the binding intensity of each antibody before and after heme exposure were plotted. A threshold that distinguishes heme-sensitive from non-sensitive antibodies was defined as the average index of binding of all native antibodies plus three standard deviations.

### Evaluation of binding kinetics

The kinetics of interaction of human monoclonal antibodies with JEV E and EDIII proteins was measured by surface plasmon resonance-based technique (BIAcore 2000, Biacore, GE Healthcare).

The proteins from JE virus were immobilized on a research grade CM5 sensor chip using amino-coupling kit (Biacore). In brief, JEV E and EDIII were diluted in 5 mM maleic acid (pH 3.85) to 10 μg/ml and injected over activated sensor surface for 7 min. HBS-EP (10 mM HEPES, pH 7.4 150 mM NaCl, 3 mM EDTA and 0.005% Polysorbat-20) was used as a running buffer. All samples during kinetics measurements were diluted in this buffer, as well.

Two-fold dilutions of heme-exposed human IgG1 antibodies (Ab21 and Rtx) (500–0.976 nM) were injected at flow rate of 20 μl/min for 360 sec. After following the dissociation, the regeneration of the binding surface was performed by 30 seconds exposure of the sensor surface to solution of 0.05 M glycine pH 12, 0.15% Triton X-100, followed by 30 sec exposure to 5M urea solution. The estimation of the kinetic constants of the interaction was done by BIAevaluation version 4.1 software (Biacore). The binding signal to the surface of the control (uncoated) flow cell was subtracted from the binding to flow cells coated with JEV proteins. Kinetics analyses of the sensorograms were performed by global analysis of the real-time binding curves data using the Langmuir binding model with a correction for drifting base-line, included in the software.

### Calculation of binding thermodynamics of monoclonal Abs to JEV proteins

Eyring’s analyses were used for calculation of the non-equilibrium thermodynamics, of the interactions of JEV envelope proteins with human antibodies exposed to heme. Kinetic measurements, as described above, were performed at 15, 20, 25, 30, and 35 °C. The reciprocal values of the temperatures in Kelvin degrees versus natural logarithm of kinetic rate constants were plotted to build Arrhenius plots. The slopes of the Arrhenius plots were calculated by using a linear regression analysis of the experimental kinetic data by using GraphPad Prism version 5 (GraphPad Prism Inc.) and substituted in the equations-


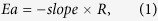


where the “slope” is equal to ∂ln(k_a/d_/∂(1/T),

*Ea* is the activation energy. The changes of enthalpy, entropy and Gibbs free energy, characterizing the association or dissociation of heme-exposed antibodies were evaluated using the following equations:


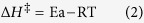



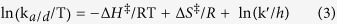



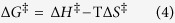


where T is the temperature in Kelvin, *k′* is the Boltzman constant and *h* is the Planck’s constant.

The equilibrium values of the thermodynamic parameters were calculated using the equations:


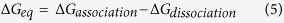



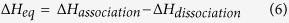



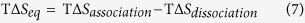


All thermodynamic parameters were determined at reference temperature of 25 °C (298.15 K).

### Focus-Reduction Neutralization Test (FRNT)

The monoclonal IgG1 (Rtx) was treated with the final heme concentration of 50 μM. FRNT was then performed with 3-fold serially diluted antibody, either heme-treated or native, incubated with approximately 50 focus forming unit (FFU) of respective virus to a 1:1 ratio at 37 °C for 1 hr. The reaction mixture was then used to infect BHK-21 cells for 1 hr at 37 °C with gentle shaking. The monolayer was then washed with PBS and overlaid with the overlay medium (1.25% methyl cellulose, 2% FBS in DMEM) for 36 hr. Foci of infected cells for each condition were detected after methanol fixation of the cell monolayer and immunostaining with an anti-JEV E antibody (4G2). The percent neutralization was calculated using the formula: 100 × (1-FFU of treatment/FFU of control) and the IC_50_ values were obtained by non-linear regression analysis with an equation of log (inhibitor) vs. normalized response in variable slope.

## Additional Information

**How to cite this article**: Gupta, N. *et al.* Neutralization of Japanese Encephalitis Virus by heme-induced broadly reactive human monoclonal antibody. *Sci. Rep.* **5**, 16248; doi: 10.1038/srep16248 (2015).

## Supplementary Material

Supplementary Information

## Figures and Tables

**Table 1:** Kinetic and equilibrium thermodynamic parameters of interaction of heme exposed human IgG_1_ Abs with JEV.

	*k*_a_ × 10^4^ mol^−1^ s^−1^	*k*_d_ × 10^−3^ s^−1^	K_D_, nM	ΔH kJ mol^−1^	TΔS kJ mol^−1^	ΔG kJ mol^−1^
mAb21-JEV E	1.22 ± 0.10	2.93 ± 0.22	240	−2.0	35.8	−37.8
mAb21-JEV EDIII	1.81 ± 0.04	2.58 ± 0.07	142	6.6	45.6	−39.1
Rtx-JEV E	1.05 ± 0.01	3.52 ± 0.12	335	16.5	53.5	−37.0
Rtx-JEV EDIII	0.83 ± 0.01	3.63 ± 0.10	435	4.7	41.0	−36.3

Values of the kinetic rate constants (*k*_a_ and *k*_d_) and equilibrium constants (K_A_ and K_D_) ± SD obtained by global analyses of sensorgrams obtained after injection of heme-exposed mAb21 and Rtx (3.9 to 500 nM) on sensor chip with immobilized JEV E and JEV EDIII. The presented values of the binding kinetics were obtained at 25 °C.
