# Supplementary material for: Neutralization of Japanese Encephalitis Virus by heme-induced broadly reactive human monoclonal antibody
Source: Sci Rep. 2015 Nov 6;5:16248. doi: 10.1038/srep16248 (PMC4635365; doi:10.1038/srep16248)
Supplement: Supplementary Information [file srep16248-s1.pdf]

Supplemental Material

Neutralization of Japanese Encephalitis Virus by heme-induced broadly reactive human monoclonal antibody

Nimesh Gupta[1,2,3,*#], Mélissanne de Wispelaere[4], Maxime Lecerf[1,2,3], Manjula Kalia[5], Tobias Scheel[6], Sudhanshu Vrati[5], Claudia Berek[6], Srinivas V. Kaveri[1,2,3], Philippe Desprès[4,7], Sébastien Lacroix-Desmazes[1,2,3], and Jordan D. Dimitrov[1,2,3,*]

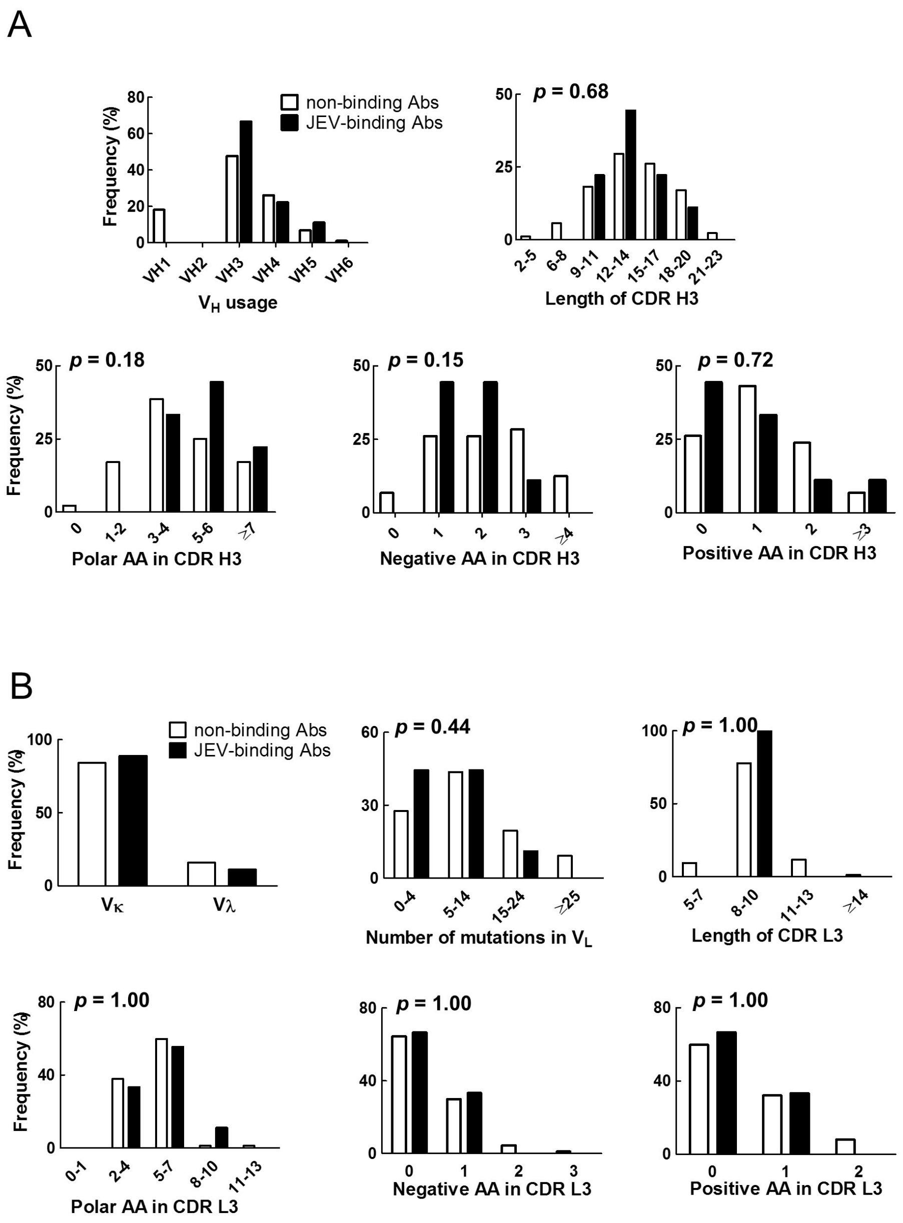

Figure S1. Analyses of gene features of variable regions of heavy and light Ig chains of heme-induced gp120 reactive monoclonal Abs. (**A**) Frequency distribution analyses of (1) gene families encoding heavy chain variable regions; (2) length of CDR H3 loop; (3) number of polar amino acid residues in CDR H3; (4) number of amino acid residues in CDR H3 with negative charge; and (5) number of acid residues in CDR H3 with positive charge. The empty bars show the distribution of VH features of Abs that did not acquire JEV E specificity after heme exposure. The filled bars represent the frequency distribution of VH characteristics of Abs that acquire binding to JEV E upon heme exposure. (**B**) Frequency distribution analyses of gene features of variable regions of light Ig (VL) chains of heme-induced JEV E specific Abs. Frequency distribution of: (1) isotype of light Ig chain; (2) number of somatic mutations

in the VL; (3) length of CDR L3; (4) number of polar amino acid residues in CDR L3; (5) number of amino acid residues in CDR L3 with negative charge; and (6) number of amino acid residues in CDR L3 with positive charge. The empty bars depicts distribution of VL characteristics of Abs that did not acquire binding to JEV after heme exposure. The filled bars depicts the distribution of VL characteristics of Abs that acquire binding to JEV upon heme exposure. Statistical analyses of frequency distributions of gene features of heavy and light Ig chains were performed by Fisher's exact test.

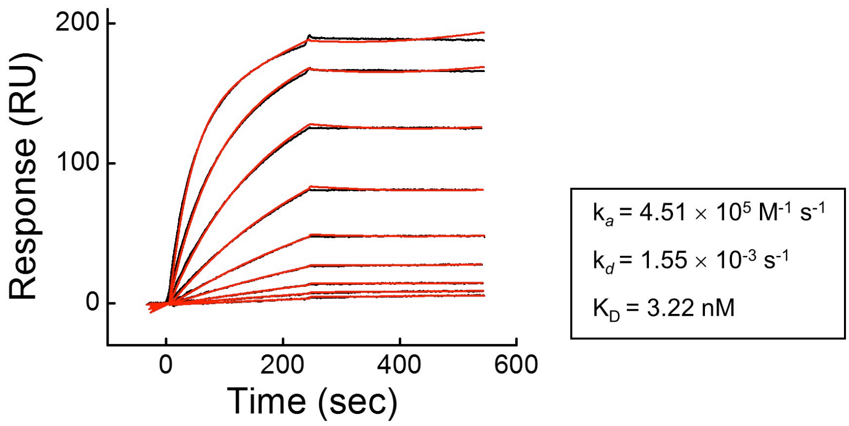

Figure S2. Real time interaction analyses of JEV-specific mouse IgG 4G2. The binding of immobilized 4G2 (immobilization density of 750 RU) to soluble JEV E protein was performed by surface plasmon resonance-based technology (Biacore). The JEV E protein was serially diluted (50 - 0.195 nM) in running buffer (HBS-EP) and each dilution of protein was injected for 4 min. The dissociation was followed for 5 min. The measurements were conducted at flow rate of 30 µl/min, at 25 °C. The graph show experimentally determined binding curves (black lines) and curves generated by globally fitting the data by BIAevaluation software (red line). The estimated kinetic constants and equilibrium affinity are presented next to the graph.

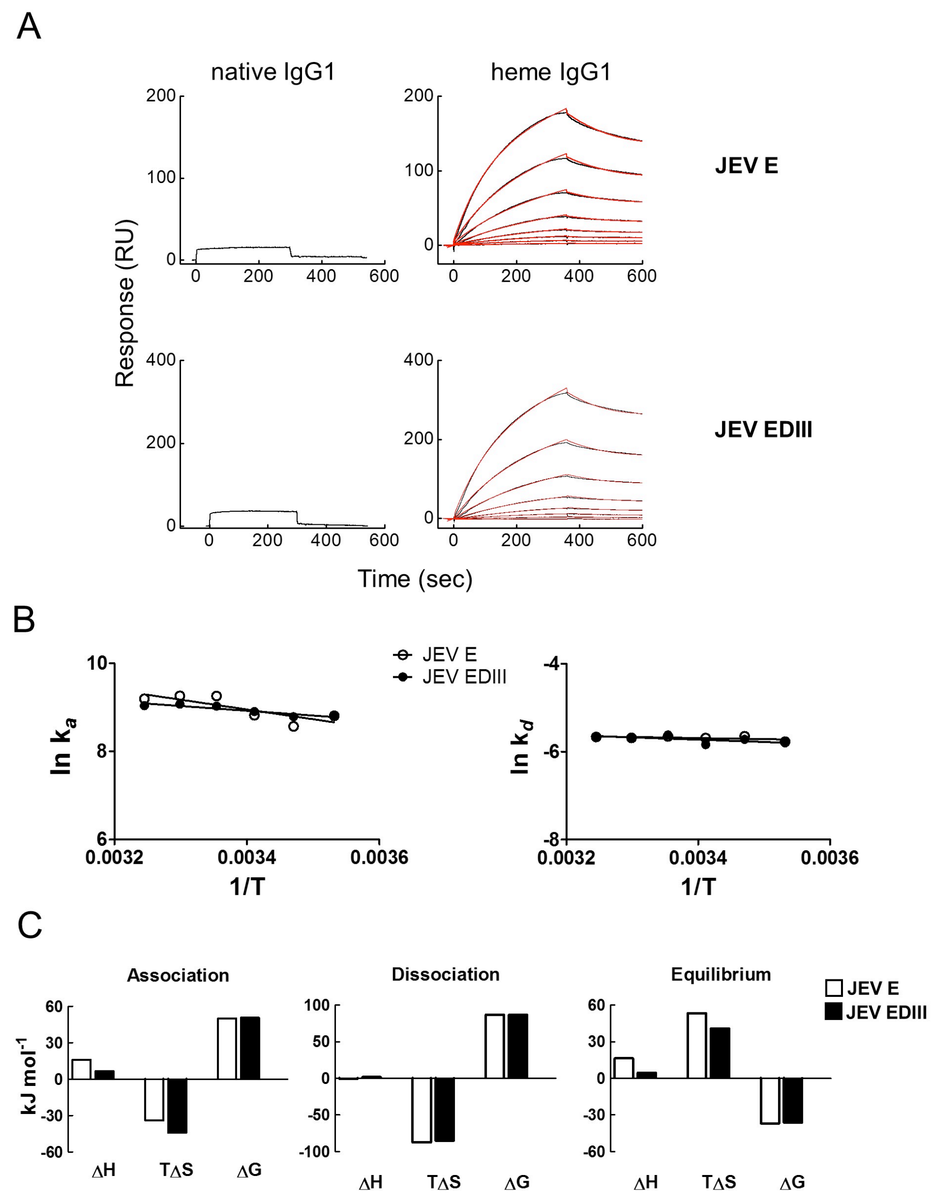

Figure S3. Kinetic and thermodynamic analyses of interaction of heme-exposed human IgG1 (Rtx) with JEV E and EDIII proteins. (**A**) Real-time interaction profiles of binding of native or heme-exposed Rtx to immobilized JEV E and EDIII proteins. The real-time interaction profiles obtained after injection of native IgG1, diluted to 500 nM are presented in the left panels. The binding profiles of heme-exposed Ab at 500, 250, 125, 62.5, 31.25, 15.63, 7.81, and 3.90 nM are presented on the right panels. The binding analyses were performed at 25°C. The graphs shows experimentally determined binding curves (black lines) and curves generated by globally fitting the data by BIAevaluation software (red line). The estimated kinetic parameters are presented on Table 1. (**B**) Arrhenius plots showing the natural logarithm values of association and dissociation rate constants of heme-exposed IgG1

obtained after interaction with JEV E (open circles) and JEV EDIII (filled circles) as a function of reciprocal values of temperature (in Kelvins). To generate these plots the kinetic rate constants were determined by global analysis of sensorgrams generated after evaluation of binding kinetics of the heme-exposed Ab with immobilized JEV proteins at varying temperatures (10, 15, 20, 25, 30, and 35°C). Linear regression analyses were applied to obtain the slopes of the temperature dependency. (**C**) Association, dissociation and equilibrium thermodynamic parameters of binding of heme-exposed IgG1 to JEV E and EDIII. Changes in the enthalpy, entropy and free energy during different phases of the interaction of heme-exposed Rtx with JEV E (white bars) and EDIII (black bars) are depicted. The changes in non-equilibrium thermodynamic parameters were evaluated by applying Eyring's analyses on the data from Arrhenius plots.
